# Decoding NETosis-associated immune dysregulation in diffuse large B-cell lymphoma through integrative multi-omics and machine learning

**DOI:** 10.3389/fcell.2026.1867660

**Published:** 2026-07-02

**Authors:** Hanbing Yao, Gangfeng Wang, Yingmin Liang, Rui Zhang

**Affiliations:** Hematology Laboratory, Xi’an International Medical Center Hospital, Xi’an, Shaanxi, China

**Keywords:** diffuse large B-cell lymphoma, immunomodulation, integrative multi-omics, machine learning, Netosis, prognostic stratification, single-cell RNA sequencing, tumor immune microenvironment

## Abstract

**Background:**

Diffuse large B-cell lymphoma (DLBCL) is a heterogeneous malignancy driven by complex immune dysregulation, yet the role of NETosis, a distinct neutrophil immune effector program, in the tumor immune microenvironment and patient prognosis remains poorly defined. This study leveraged an integrative multi-omics and machine learning framework to elucidate the immunological implications of NETosis-related genes (NETosis-RGs) in DLBCL.

**Methods:**

NETosis-RGs were identified from transcriptomic profiles of DLBCL versus control tissues (GSE32018). Prognostic candidates were screened by univariate Cox regression and modeled using five machine learning algorithms, with the Random Survival Forest (RSF) model selected for risk stratification. Bulk and single-cell transcriptomic data were integrated to resolve the immune landscape, while ceRNA networks and m^6^A regulator analyses were performed to characterize post-transcriptional and epigenetic layers. Chemotherapy sensitivity was predicted to explore therapeutic vulnerabilities.

**Results:**

The RSF-based model, incorporating five key NETosis-RGs (AKT1, SLC25A37, FPR2, TLR7, F3), robustly stratified patients into high- and low-risk groups exhibiting systematically distinct immune architectures—spanning differential immune cell infiltration, stromal/immune scores, and immune checkpoint expression. Single-cell transcriptomic analysis further revealed that these prognostic genes were predominantly expressed in monocytes and M1 macrophages and displayed dynamic regulation along myeloid/macrophage differentiation trajectories, suggesting their role as potential modulators of the immunosuppressive-to-inflammatory transition within the DLBCL microenvironment. Complementary analyses revealed multi-layered regulatory mechanisms, including m^6^A modification patterns and lncRNA–miRNA–mRNA ceRNA axes.

**Conclusion:**

By integrating multi-omics data with machine learning, this study delineates a novel NETosis-centered immune signature that refines prognostic stratification and provides mechanistic insights into monocyte/macrophage-mediated immune dysregulation in DLBCL. The findings offer a framework for future immunomodulatory biomarker development and the development of precision therapeutic strategies.

## 1 Introduction

Diffuse large B-cell lymphoma (DLBCL) is the most common aggressive B-cell malignancy, with approximately 150,000 new cases annually worldwide (Sehn and Salles, 2021; Barraclough et al., 2024; Shi et al., 2023). Although 50%–60% of patients achieve durable remission following first-line chemoimmunotherapy, 30%–40% relapse—typically within 2 years—and approximately 10% develop primary refractory disease (Chan et al., 2024; Kang et al., 2020). Because patients with similar clinicopathological features often experience markedly divergent outcomes, there is a pressing need to dissect the molecular and cellular determinants underlying this heterogeneity, particularly those operating within the tumor immune microenvironment.

Neutrophils, as essential innate immune effector cells, are increasingly recognized not only as antimicrobial first responders but also as active participants in tumor immunity (Brostjan and Oehler, 2020; Pérez-Figueroa et al., 2021; Barnado et al., 2016; Nie et al., 2019). A defining functional program of neutrophils is NETosis—a regulated process culminating in the extrusion of neutrophil extracellular traps (NETs) composed of decondensed chromatin, histones, and granular proteins (Brostjan and Oehler, 2020). Initially described as a host-defense mechanism that immobilizes and eliminates pathogens, NETosis has more recently been implicated in both pathological inflammation and tumor progression (Barnado et al., 2016; Nie et al., 2019; Qi et al., 2023). In DLBCL, tumor-associated neutrophils and elevated NET burden in plasma or tumor tissue have been linked to adverse prognosis (Shi et al., 2023). Mechanistically, lymphoma cells can directly induce NETosis, and the resultant NETs may paradoxically foster a microenvironment conducive to tumor growth and drug resistance (Qi et al., 2023). These observations position NETosis as a compelling avenue for characterizing immune dysregulation and identifying novel immunomodulatory targets in DLBCL.

To date, however, comprehensive analyses linking NETosis-associated molecular programs to the broader immune landscape and patient stratification in DLBCL are scarce. Here, we harnessed an integrative multi-omics framework—combining bulk and single-cell transcriptomics with machine learning—to systematically decode the immunological role of NETosis-related genes (NETosis-RGs). Our study constructs a robust immune-informed prognostic model, resolves the heterogeneity of the tumor immune microenvironment across risk strata, and characterizes the cellular context and regulatory networks through which key NETosis-RGs may modulate DLBCL progression. This work aims not only to refine prognostic stratification but, more fundamentally, to provide a mechanistic foundation for future immunomodulatory strategies in this clinically heterogeneous disease.

## 2 Materials and methods

### 2.1 Data collection

This study utilized the Gene Expression Omnibus (GEO) (https://www.ncbi.nlm.nih.gov/gds) database. The study analyzed RNA-seq data from GSE32018 (DLBCL and control lymph node samples) for gene identification, GSE10846 (414 DLBCL samples with clinical and survival data) for prognostic model building, GSE11318 (199 DLBCL lymph node tumor tissue samples) for model validation, and GSE182435, a single-cell immune repertoire sequencing (scVDJ-seq) dataset (4 DLBCL lymph node tumor tissue samples), and GSE182434, a single-cell RNA sequencing (scRNA-seq) dataset (7 DLBCL lymph node and 1 tonsillitis samples), for expression analysis. GSE182435 and GSE182434 constitute subseries of GSE182436 and were jointly used to perform all single-cell analyses in this study, using 69 NETosis-related genes from the literature. (Qi et al., 2023).

### 2.2 Difference expression gene analysis

In GSE32018, differential expression analysis was conducted using the limma package (v3.40.6) (Ritchie et al., 2015) to obtain the differentially expressed genes (DEGs). Visualizations of DEGs (|log2FC| > 0.5 and P. adjust <0.05), including volcano plots and heat maps, were generated with the ggplot2 package (v 3.3.0) (Gustavsson et al., 2022) and the Pheatmap package (v 0.7.7) (Gustavsson et al., 2022). The intersection of the DEGs and the NETosis-Related was identified to yield the differentially expressed NETosis-Related DEGs (NETosis-RGs), and visualized by using the ggvenn package (v 0.1.10) (Pelikan et al., 2022).

### 2.3 Functional annotation of the NETosis-RGs

To identify related functions and pathways, NETosis-RGs underwent enrichment analysis for Gene Ontology (GO) and Kyoto Encyclopedia of Genes and Genomes (KEGG) utilized with the clusterProfiler package (P.adjust <0.05) (v 3.8.1) (Wu et al., 2021). And Protein-Protein Interaction (PPI) networks, with the criterion of median PPI score >0.4, were constructed using the STRING (http://string-db.org) database and visualized using Cytoscape software (v 3.10.1) (Shannon et al., 2003). In addition, Gene Set Enrichment Analysis (GSEA) was performed to explore immune-related pathways, utilizing the clusterProfiler package (v 4.14.6) with immune gene sets obtained from the Molecular Signatures Database (MSigDB).

### 2.4 Feulgen DNA staining

We gathered samples of newly diagnosed pathological specimens of diffuse large B-cell lymphoma from 2023 to 2025 at the Medical Center (*The ethics committee of the institution has approved this research, No. GJYX-XY-2023-003*). Paraffin sections were dewaxed in xylene for 20 min, then replaced with fresh xylene and dewaxed for another 20 min. Then dehydrate anhydrous ethanol, 90%, 80%, and 70% ethanol gradient for 2 min each time. After dehydration, the slices were hydrolyzed in a preheated weak acid solution at 60 °C for 8 min, then removed and slightly moistened with the weak acid solution at room temperature, and washed again with distilled water three times for 1 min each. Using a PAP Pen, circle the tissue sample, drop 50 μl of Schiff reagent on the section to ensure complete coverage of the tissue sample, stain protected from light for 90 min without washing, and then rinse the section directly in 1X sulfite solution twice for 2 min each time. Afterwards, immerse in a solid green dyeing solution for 45 s, and rinse with distilled water to remove excess dyeing solution. The tablets were sealed with neutral gum and then observed and photographed under a microscope.

### 2.5 Immunofluorescence imaging

The collected paraffin pathological sections were immersed in xylene three times, each time for 5 min. Then, they were infiltrated with anhydrous ethanol three times, each time for 5 min, followed by 95% ethanol once for 5 min, and 70% ethanol once for 5 min. Finally, they were rinsed with double-distilled water for 5 min to remove paraffin and adjust the sample’s water content. Antigen repair was performed with citrate repair solution to restore the antigen conformation. Then, the samples were blocked with 10% goat serum at room temperature for 1 h to reduce background staining. The target tissue was enclosed using a PAP Pen, and the diluted primary antibody was added dropwise and incubated overnight at 4 °C. TBST buffer was washed 3 times for 10 min each time. The secondary antibody was then incubated with the fluorescently labeled solution for 1 h at room temperature. DAPI working solution was added dropwise and stained at room temperature for 10 min, labeling the cell nucleus. The TSA reagent was then incubated for multiple rounds to enhance the fluorescence signal. The sealing liquid was applied with an anti-fluorescence agent to avoid bubble formation, and then observed and photographed under a confocal microscope. Colocalization analysis was performed using the Plot-Profile algorithm of ImageJ (N.I.H., Bethesda, MD) (Duan et al., 2018).

### 2.6 Construction of a prognostic model

To refine gene selection, a univariate Cox proportional hazards regression (Cox) analysis was first performed on NETosis-RGs in the training cohort. We employed the Schoenfeld residual-based test to evaluate the proportional hazards (PH) assumption for each candidate gene. In this test, P > 0.05 indicates that the PH assumption is not violated. Accordingly, only genes that simultaneously met two criteria were retained (Sehn and Salles, 2021): PH assumption satisfied (Schoenfeld test P > 0.05), and (Barraclough et al., 2024) significance in univariate Cox regression (P < 0.05). This process yielded five prognosis-associated candidate genes (SLC25A37, TLR7, AKT1, FPR2, and F3). Protein expression patterns of these genes were further examined using the Human Protein Atlas (HPA) database to confirm biological relevance (see method Section 2.12 for details). The selected genes were then used as input features for constructing prognostic models with five machine learning algorithms: least absolute shrinkage and selection operator (LASSO) regression, ridge regression, elastic net regression, random survival forest (RSF), and extreme gradient boosting (XGBoost). The glmnet package (v4.1.8) (Li et al., 2022) was used to build the LASSO, ridge, and elastic net models, with optimal penalty parameters (log λ) determined via cross-validation. The ranger package (v0.17.0) and xGboost package (v1.7.11.1) were applied to develop the RSF and XGBoost models, respectively, with the parameter family set to Cox. Model performance was evaluated by concordance index (C-index) and time-dependent receiver operating characteristic (ROC) analysis at 1-, 3-, and 5-year survival intervals. Feature importance metrics were computed for RSF and XGBoost models to quantify the relative contribution of each gene to survival prediction. The best-performing model was selected for downstream risk score calculation and survival stratification. We employed the ranger package (v0.17.0) to develop a random survival forest (RSF) model, configuring the primary parameters as: num-trees = 1,000, mtry = 3, min.node.size = 5. Model performance was evaluated using the concordance index (C-index) and time-dependent ROC curves. The risk score cut-off value was defined as the median risk score of patients in the training set. In the external validation cohort (GSE11318), we applied the same risk score formula and the median cut-off value obtained from the training set without modification to classify patients into high-risk and low-risk groups. To prevent overfitting and data leakage, we adhered to standard procedures during model development: feature selection (univariate Cox regression) was restricted to the training set (GSE10846), parameter tuning was performed via internal cross-validation, and final model evaluation alongside cut-off determination was conducted separately on the internal training and external validation sets.

### 2.7 Independent prognostic analysis

To identify independent prognostic factors, clinical variables (age, gender, TNM stage, and tumor stage) and the risk groups were included in the univariate Cox analysis. The Cox models were tested for the PH assumption, and clinical factors (p < 0.01and PH > 0.05) were selected. Concurrently, a multivariate Cox model was constructed, incorporating clinical variables, and a stepwise algorithm was utilized to passively select the final independent prognostic factors. Based on the aforementioned results, a nomogram was constructed using the rms package (v 5.1-4) (Pan et al., 2021). A calibration curve for the nomogram was plotted. Furthermore, significant differences in risk scores among different clinical characteristics were compared.

### 2.8 Validation of the prognostic model

The robustness of the prognostic models was further validated using the GSE11318 dataset as an independent external cohort. For each patient, risk scores were computed according to the five prognostic algorithms derived from the training phase. Patients were then stratified into high- and low-risk groups based on the corresponding algorithm-specific cut-off values. To assess model performance, risk score distributions and survival status plots were generated, followed by time-dependent receiver operating characteristic (ROC) analysis to evaluate predictive accuracy at 1-, 3-, and 5-year time points. Kaplan–Meier (KM) survival analyses were conducted to compare overall survival between high- and low-risk groups. Additionally, heatmaps were constructed to visualize the expression profiles of the prognostic genes within GSE11318.

### 2.9 Gene set enrichment analysis (GSEA)

In GSE10846, the limma package (v 3.40.6) (Ritchie et al., 2015) was used for differential analysis between high- and low-risk groups, and genes were sorted based on the fold change in expression difference. Subsequently, GSEA enrichment analysis was performed.

### 2.10 Immune infiltration analysis

To delve deeper into how prognostic NETosis-RGs contribute to immune infiltration, the estimate analysis of immunedeconv package (v 2.1.0) (Sturm et al., 2019) was applied. These scores underwent Wilcoxon testing, with the findings depicted in box plots. Additionally, the cibersort analysis within the immunedeconv package (v 2.1.0) (Sturm et al., 2019) facilitated a comparative examination of immune cell frequencies across risk groups, with the disparities in these frequencies also evaluated using the Wilcoxon test. Spearman analysis was used to correlate immune cell abundance with risk scores and prognostic gene expression.

### 2.11 Analysis of prognostic genes in relation to particular factors

To examine B-cell biomarkers in DLBCL, we compared their expression in risk groups using the Wilcoxon test in GSE10846, followed by Spearman analysis. Visualization was done with ggplot2 (v 3.3.0) box plots (Gustavsson et al., 2022). Comparisons and visualizations of 20 m^6^A regulatory elements were performed similarly. In GSE10846, differences in immune checkpoint inhibitors (ICIs) for both groups were analyzed, with ggplot2 package (v 3.3.0) (Gustavsson et al., 2022) used to plot box charts showing these differences via Wilcoxon method. Spearman analysis was also performed to correlate risk scores with immune checkpoints.

### 2.12 Analysis of chemotherapy drug sensitivity

For the prediction of effective therapeutic agents, the GSE10846 was analyzed to contrast the responsiveness to anticancer medications between patients classified as high-risk and those as low-risk. The Inhibitory Concentration 50 (IC50) values for these medications were sourced from the Genomic Data Sharing Consortium database (GDSC) database. Utilizing the oncoPredict package (v 0.2) (Maeser et al., 2021), IC50 calculations were performed, and the Wilcoxon test was applied to discern two drugs with the lowest mean IC50 (under 1) and the most pronounced significance.

### 2.13 Prognostic gene HPA analysis and construction of regulatory networks

To confirm biomarker protein expression in Lymphoma and control samples. Immunohistochemistry results of the biomarkers were obtained from the Human Protein Atlas (HPA) database (https://www.proteinatlas.org/) to confirm the expression of the biomarkers/proteins. Utilizing the multiMiR package (v 1.1.0) (Ru et al., 2014), validated miRNAs associated with prognostic NETosis-RGs were retrieved from the miRtarbase database (https://mirtarbase.cuhk.edu.cn/). By employing the multiMiR package (v 1.1.0) (Ru et al., 2014)within the mirtarbase database (https://mirtarbase.cuhk.edu.cn/), miRNAs and lncRNAs related to validated prognostic NETosis-RGs were identified. Subsequently, a ceRNA regulatory network was constructed using Cytoscape (v 3.10.1) (Shannon et al., 2003).

### 2.14 Single-cell sequencing analysis

Download 8 DLBCL samples from GSE182434, filter single-cell RNA-seq data with Seurat’s CreateSeuratObject (v 4.2.0) (Satija et al., 2015), excluding genes detected in <3 cells or with <200 genes per cell (min.features = 200, min.cells = 3). Remove cells with features outside 200–4000 range or counts >20,000. Use PercentageFeatureSet to filter for cells with mitochondrial gene ratio <7.5%, yielding a set of viable cells.

Then, the highly variable genes selected above were normalized using the ScaleData function, followed by the execution of Principal Component Analysis (PCA). Subsequently, linear dimensionality reduction was performed using JackStraw and ScoreJackStraw. The information represented by each principal component (percentage of variance) was sorted by percentage, and a PCA inflection point plot and a scree plot were plotted to select significant principal components. The Uniform Manifold Approximation and Projection (UMAP) algorithm was used to perform dimensionality reduction analysis on the samples. Annotations for the obtained cells were made using the inherent labels from the original dataset. And the expression levels of prognostic genes in different cells were displayed using a bubble plot. To assess intercellular communication, an analysis of cell signaling was conducted.

### 2.15 Pseudotime trajectory construction for monocytes/macrophages

The Find VariableFeatures function was used to identify genes with high expression variability among monocytes/macrophages (selection.method = vst, nfeatures = 2000). The ScaleData function was applied to normalize the aforementioned results, followed by conducting PCA. Linear dimensionality reduction was performed using JackStraw and ScoreJackStraw. The information represented by each principal component (percentage of variance) was sorted by percentage, and a PCA inflection point plot and scree plot were plotted to select significant principal components. Dimensionality reduction was further pursued using the t-Distributed Stochastic Neighbor Embedding (tSNE) algorithm. Subtype annotations for monocytes/macrophages were made based on biomarkers identified in the literature. High-variable genes within monocytes/macrophages were employed as feature genes, and through the processes of dimensionality reduction and cell ordering using the DDRTree method, a cell pseudotime trajectory was constructed.

### 2.16 Statistical analysis

Statistical analyses were conducted using R software (version 4.1.0). Differences in immune cell abundance, immune checkpoint expression, m^6^A regulator expression, and drug sensitivity across risk groups were evaluated using the Wilcoxon rank-sum test. To control the false discovery rate (FDR) arising from multiple comparisons, all P values were adjusted using the Benjamini-Hochberg method. Statistical significance was defined as an adjusted P value <0.05. Similarly, in correlation analysis (e.g., Spearman correlation between risk score and immune checkpoints), we also reported the adjusted P values.

## 3 Results

### 3.1 Identification and validation of NETosis-RGs in DLBCL through integrated bioinformatics and immunohistochemical analysis

**FIGURE 1:**
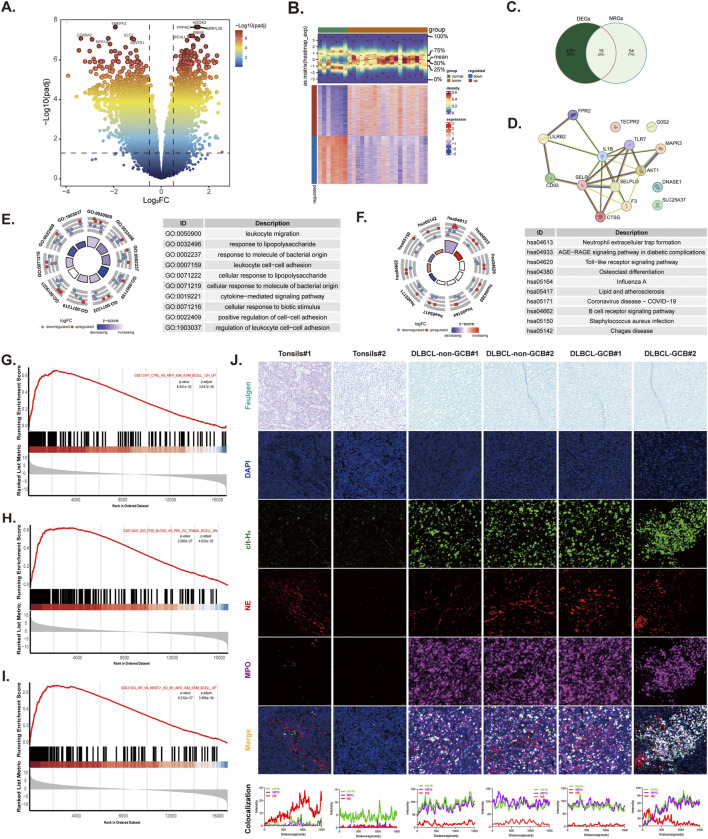
Identification and functional characterization of NETosis-RGs in DLBCL. **(A,B)** Volcano plots and heatmap showing the total of 4,276 DEGs, with2,186 upregulated and 2,090 down-regulated, were screened in GSE32018. Visualizations of DEGs |log2FC|>0.5 and P-adjust<0.05. **(C)** Venn diagram showing the intersection of DEGs with NETosis-RGs to obtain differentially expressed genes. **(D)** Protein-Protein Interaction (PPI) networks demonstrate NETosis-RGs correlations, the criterion of median PPI score >0.4. TECPR2, G0S2, SLC25A37, and DNASE1 showed no significant interactions under this criterion and are displayed separately. **(E)** Left: Radar graph showing Gene Ontology (GO)-derived gene function analysis. P-adjust<0.05. Right: Table showing GO-enriched terms information. **(F)** Left: Radar graph showing gene function analysis derived from Kyoto Encyclopedia of Genes and Genomes (KEGG), P. adjusted<0.05. Right: table showing information on KEGG-enriched terms. **(G–I)** GSEA enrichment plots showing enrichment of immune-related pathways for NFATC1 knockout versus wild-type B cell anti-IgM stimulation, anti-IgM-stimulated B cells at 12 h, and IgD^+^ blood B cells versus pre-germinal center tonsil B cells. **(J)** Representative Feulgen DNA staining showing DNA in magenta with light green counterstain in paraffin-embedded DLBCL tissue sections and immunofluorescence staining demonstrates the expression of citrullinated histone H3 (citH3, green), neutrophil elastase (NE, red), and myeloperoxidase (MPO, purple). Nuclei were counterstained with DAPI (blue). Colocalization analysis was performed using the Plot Profile algorithm. Images were acquired using a confocal microscope. Scale bar = 100 μm.

We first analyzed the GSE32018 dataset and identified 4,276 differentially expressed genes (DEGs), comprising 2,186 that were upregulated and 2,090 that were downregulated (Figures 1A,B). To focus on genes potentially involved in neutrophil extracellular trap formation, we intersected these DEGs with a curated list of NETosis-RGs, yielding 15 NETosis-related candidates (NETosis-RGs): CTSG, MAPK3, AKT1, SELPLG, CD93, SLC25A37, DNASE1, IL1B, FPR2, TLR7, SELP, TECPR2, F3, LILRB2, and G0S2 (Figure 1C). To explore their functional relationships, we constructed a protein–protein interaction (PPI) network, which revealed strong associations among these NETosis-RGs, including AKT1–MAPK3, IL1B–FPR2, and CD93–LILRB2 (Figure 1D). The network suggested that these genes may cooperate in coordinated immune responses, prompting us to perform functional enrichment analyses. Gene Ontology (GO) analysis demonstrated that these genes were predominantly involved in leukocyte migration, responses to lipopolysaccharide (LPS) and bacterial-derived molecules, leukocyte cell–cell adhesion, cytokine-mediated signaling, and regulation of leukocyte adhesion (Figure 1E). These functions point toward an immune-activated tumor microenvironment. Consistent with this, KEGG pathway enrichment indicated significant involvement in NET formation, as well as pathways linked to inflammatory and infectious processes, including AGE–RAGE signaling in diabetic complications, Toll-like receptor signaling, osteoclast differentiation, influenza A mechanisms, lipid metabolism in atherosclerosis, COVID-19 pathophysiology, B cell receptor signaling, *Staphylococcus aureus* infection, and Chagas disease (Figure 1F). Given the enrichment of B cell–related pathways, we further explored immune signaling using gene set enrichment analysis (GSEA). This analysis revealed significant enrichment in signatures such as “NFATC1 KO vs. WT B cell anti-IgM stimulation”,“Anti-IgM-stimulated B cell 12H″ and “IgD^+^ blood vs. pre-GC tonsil B cell” suggesting a potential link between NETosis-related activity and B cell dysfunction (Figures 1G-I, Supplementary Figure S1). To validate these bioinformatics findings in clinical samples, we performed immunofluorescence (IF) staining for three core markers associated with neutrophil extracellular traps (NETs)—citrullinated histone H_3_ (Cit-H_3_), neutrophil elastase (NE), and myeloperoxidase (MPO)—along with Feulgen staining for DNA and DAPI immunofluorescence staining (Figure 1J). In diffuse large B-cell lymphoma (DLBCL) tissues, CitH3, NE, and MPO exhibited markedly stronger cytoplasmic and nuclear staining in infiltrating immune cells compared with normal tonsil tissue. Co-localization analysis revealed distinct co-localization signals between CitH3 and MPO, which is consistent with the predicted increase in NET formation. (Figure 1J). Taken together, these results integrate bioinformatics predictions with histological validation, establishing that DLBCL is characterized by an active, NETs-enriched immune microenvironment potentially linked to altered B cell function.

### 3.2 Construction and evaluation of a machine learning-based prognostic model using NETosis-RGs

**FIGURE 2:**
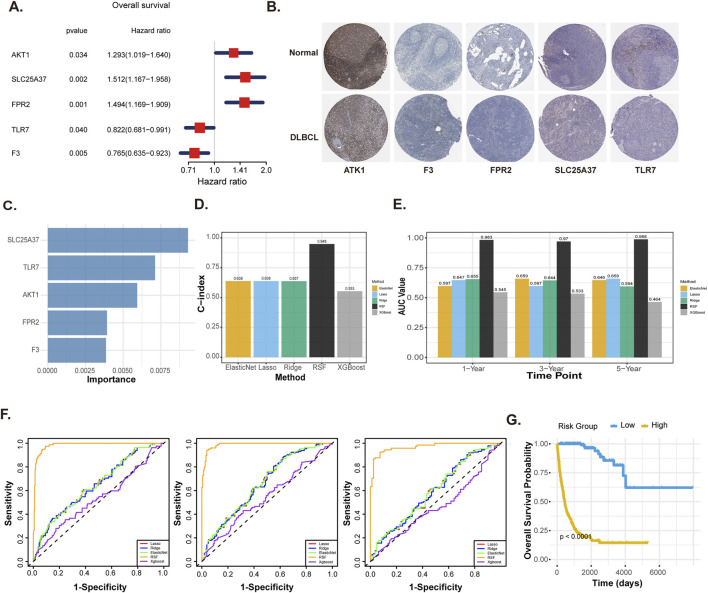
RSF-based prognostic modeling of NETosis-RGs outperforms alternative approaches in DLBCL. **(A)** Prognostic gene screening by univariate Cox regression analysis (p < 0.05). **(B)** Immunohistochemical expression of the five prognostic biomarkers (SLC25A37, TLR7, AKT1, FPR2, and F3) from the Human Protein Atlas (HPA) database. **(C)** Feature importance of the five prognostic genes in the random survival forest (RSF) model. **(D)** Comparison of concordance index (C-index) among different prognostic models, highlighting the superior predictive accuracy of the RSF model. **(E)** Time-dependent area under the curve (AUC) for 1-, 3-, and 5-year overall survival (OS), showing higher predictive performance of RSF compared with other models. **(F)** Receiver operating characteristic (ROC) curves of all models, confirming the strongest discriminative ability of RSF. **(G)** Kaplan–Meier survival curves stratified by RSF-derived risk scores, where high-risk patients exhibited significantly poorer OS than low-risk patients.

In the training set, we first performed univariate Cox regression analysis for 15 NETosis-RGs. Prior to the primary analysis, we used the Schoenfeld residuals method to test the proportional hazards (PH) assumption for each gene. The test results showed that all 15 genes satisfied the PH assumption (all P > 0.05), indicating that no genes were excluded based on this criterion. Subsequently, a univariate Cox regression analysis revealed five NETosis-RGs significantly associated with prognosis in the training cohort (SLC25A37, TLR7, AKT1, FPR2, F3). (Figure 2A). To provide supportive protein-level evidence for their biological relevance, we examined protein expression patterns in the Human Protein Atlas (HPA) database, where all five genes showed detectable expression in lymphoid or tumor tissues. (Figure 2B). These five prognostic genes were then used as candidate features to construct prognostic models using five machine learning algorithms: least absolute shrinkage and selection operator (LASSO) regression, ridge regression, elasticnet regression, random survival forest (RSF), and extreme gradient boosting (XGBoost). The tuning processes for LASSO, ridge, and elastic net models, including the selection of optimal penalty parameters (log λ), are shown (Supplementary Figures S2A-F), while the relative feature importance of each gene in the XGBoost model is presented (Supplementary Figure S2G). Among these, the RSF model demonstrated the best prognostic performance. Feature importance analysis in the RSF model highlighted the relative contribution of each gene (Figure 2C), suggesting a non-uniform impact on survival prediction. Quantitatively, the RSF model achieved the highest concordance index (C-index) of 0.948, substantially outperforming the other models (Figure 2D). Time-dependent receiver operating characteristic (ROC) analysis further confirmed its superiority, with AUC values at 1-, 3-, and 5-year survival all exceeding 0.97, whereas other models displayed lower predictive accuracy (Figure 2E). The ROC curves consistently showed the RSF model achieving the greatest classification performance across all time points (Figure 2F). Based on RSF-derived risk scores, patients were stratified into high- and low-risk groups using the median score as the cutoff. Kaplan–Meier survival analysis revealed a highly significant separation in overall survival between the two groups (P < 0.001), demonstrating that the RSF-based risk model has robust discriminatory ability for predicting survival outcomes in DLBCL (Figure 2G; Supplementary Figures S2H-J). The RSF model effectively stratified patients into high-risk and low-risk groups; Kaplan-Meier analysis revealed a statistically significant difference in overall survival (OS) between the two groups (P < 0.001), indicating significantly poorer overall survival for the high-risk group.

### 3.3 External validation of the prognostic model

**FIGURE 3:**
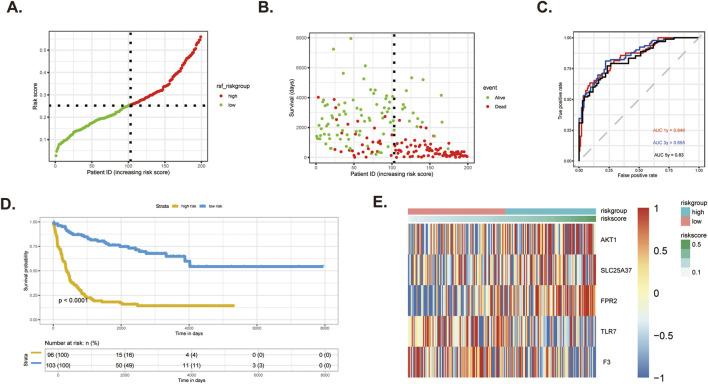
External validation of multiple prognostic NETosis-RG models in DLBCL patients. **(A)** Distribution of RSF-derived risk scores across patients, ranked by increasing risk score. High-risk and low-risk groups are indicated. **(B)** Survival days and status of each patient corresponding to the risk scores in **(A)**; shorter survival times are concentrated in the high-risk group. **(C)** Time-dependent receiver operating characteristic (ROC) curves evaluating predictive performance of the RSF model at 1-, 3-, and 5-year overall survival. **(D)** Kaplan–Meier survival curves stratified by RSF-derived risk scores. The number at risk for high- and low-risk groups is shown below each curve. **(E)** Heatmap showing the expression patterns of the five prognostic NETosis-RGs across patients, with samples ordered according to their risk score.

To further assess the robustness of the prognostic models, the GSE11318 dataset served as an independent-external validation cohort, applying the same model and cut-off value. Risk scores were calculated for each patient using the five prognostic algorithms (Figure 3A; Supplementary Figures S3A-D) and subsequently applied to stratify individuals into high- and low-risk groups. For visualization, patients were ranked by their respective risk scores along the x-axis, with survival days plotted on the y-axis. In the RSF model, a pronounced downward clustering toward the bottom-right corner was observed, indicating that patients with higher predicted risk scores consistently exhibited shorter survival times compared with other models (Figure 3B; Supplementary Figures S3E-H), thereby demonstrating the strongest risk stratification capability. Predictive performance was further evaluated by generating ROC curves and Kaplan–Meier (KM) survival analyses for all models. Across all evaluated time points, the RSF model achieved the highest AUC values (1-year = 0.846, 3-year = 0.855, 5-year = 0.833) (Figure 3C; Supplementary Figures S3I-L) and showed the most significant separation between high- and low-risk groups in KM analyses (Figure 3D; Supplementary Figures S3M-P), underscoring its superior prognostic accuracy and discriminative power. Additionally, we visualized the expression profiles of the five prognostic genes in the GSE11318 cohort using a heatmap (Figure 3E). Distinct expression patterns were observed between the high- and low-risk groups, which were consistent with the expected trends from the training phase, further supporting the robustness of these genes as key components of the RSF model. Collectively, these results confirm that the RSF model not only excels in risk prediction but also maintains stable and reliable performance across independent datasets.

### 3.4 Ability to assess risk models and clinical factors as independent prognostic factors

**FIGURE 4:**
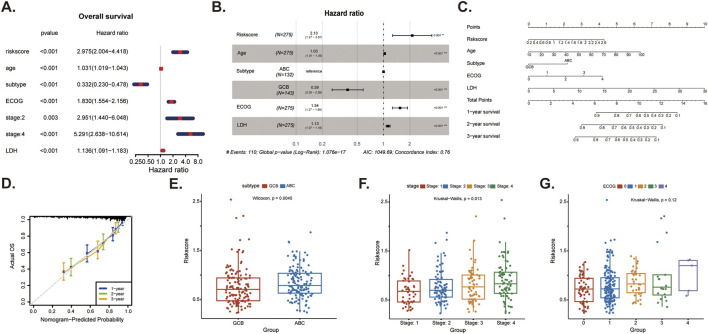
Independent prognostic factors. **(A)** Forest plot for one-factor cox regression analysis. **(B)** Forest plot of multifactorial independent prognostic model. **(C,D)** Construction of prognostic model line graphs and calibration curves to assess gender, N stage and high and low risk groups. **(C)** Line diagram model. **(D)** Calibration curve for line graphs. **(E–G)** Rank-sum test of risk scores in clinical characteristics. **(E)** Differences in risk scores between subtypes, Wilcox test. p < 0.01; PH: p > 0.05. **(F)** Differences in risk scores between tumor sub-periods. Kruskal-Wallis test. p < 0.01; PH: p > 0.05. **(G)** Differences in risk scores between ECOG subgroups. Kruskal-Wallis test. p < 0.01; PH: p > 0.05.

To evaluate the relationship of risk models and clinical factors with DLBCL, a prognostic analysis was conducted. Univariate Cox regression analysis showed that the risk score, age, disease subtype, ECOG performance status, tumor stage, and LDH score were all correlated with the prognosis of DLBCL (Figure 4A). Multivariate Cox regression analysis confirmed that all factors, except for tumor stage, were significantly associated with the prognosis of DLBCL (Figure 4B). A nomogram model was constructed using gender, N-stage, and high/low-risk groupings. The total score, obtained by summing the individual factors, was used to predict the 1-, 2-, and 3-year survival rates of DLBCL patients. The higher the score, the lower the survival rate (Figure 4C). Additionally, calibration curves showed that the model accurately predicted 1-, 2-, and 3-year survival rates, validating its effectiveness as a prognostic tool (Figure 4D). And it was found that the ABC subtype had a significantly higher risk score. The risk score also increased with higher tumor staging in the samples. However, no notable variance in risk scores was observed among various ECOG groups (Figures 4E–G).

### 3.5 Functional and immune landscape characterization based on RSF-derived risk stratification

**FIGURE 5:**
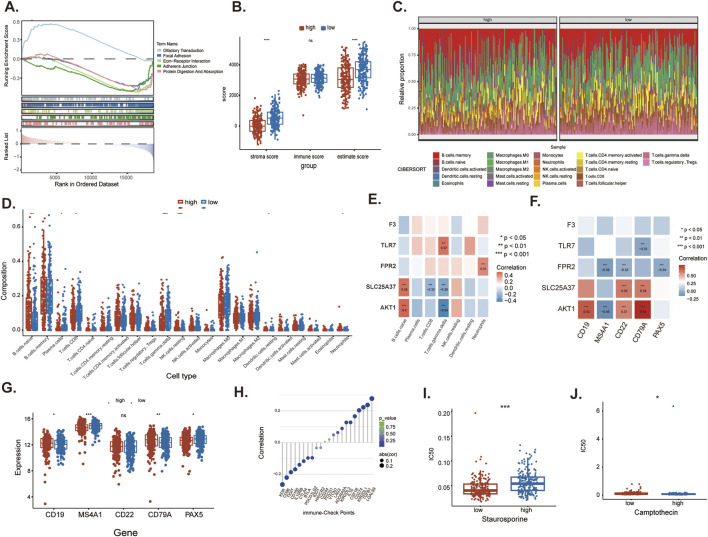
Immune landscape and drug sensitivity analysis in high- and low-risk DLBCL patients. **(A)** Gene set enrichment analysis (GSEA) showing pathways enriched in high-versus low-risk groups. **(B)** Comparison of immune-related scores, including risk score, stromal score, immune score, and ESTIMATE score, between high- and low-risk groups. **(C)** CIBERSORT analysis of immune cell composition in high- and low-risk groups. **(D)** Dot plot of immune cell proportions between high- and low-risk groups. **(E)** Heatmap showing expression of five prognostic NETosis-RGs across different immune cell types. **(F)** Heatmap showing expression of five prognostic NETosis-RGs across B cell marker genes. **(G)** Dot plot of B cell marker expression in high- and low-risk groups. **(H)** Lollipop plot of immune checkpoint gene expression between high- and low-risk groups. **(I,J)** Predicted IC50 values for chemotherapeutic drugs in high-versus low-risk patients. * = *P*. adjust<0.05, ** = *P*. adjust<0.01, *** = *P*. adjust<0.001.

To further elucidate the biological mechanisms underlying RSF-derived risk groups, we performed a comprehensive set of functional and immunogenomic analyses. Gene set enrichment analysis (GSEA) revealed distinct pathway enrichments between high- and low-risk groups (Figure 5A), indicating differential activation of stromal processes. Consistently, ESTIMATE analysis demonstrated that high-risk patients exhibited significantly altered stromal, immune, and overall ESTIMATE scores compared with low-risk patients (Figure 5B). Further immune cell profiling using CIBERSORT highlighted significant differences in the abundance of multiple immune cell types between risk groups (Figures 5C,D; Supplementary Figures S4A,B). Furthermore, CIBERSORT analysis revealed significant differences in the proportions of M2 macrophages and tumor-associated macrophages (TAMs) between the high-risk and low-risk groups. Compared with the low-risk group, the high-risk group exhibited higher levels of infiltration of M2 macrophages and TAMs. M2 macrophages are characterized by immunosuppressive and tumor-promoting functions, while TAMs in the tumor microenvironment often display an M2-like phenotype. Therefore, the enrichment of M2 macrophages and TAMs in the high-risk group may reflect a more pronounced immunosuppressive microenvironment, which is consistent with the poorer prognosis and differences in immune checkpoint expression profiles observed in this group (Figure 5C). Correlation analysis between the five prognostic genes and the differential immune cell subsets identified in CIBERSORT revealed strong associations, particularly with B cell-related populations, including AKT1 and SLC25A37 (Figure 5E). Focusing on these B cell-related markers, heatmaps and boxplots confirmed distinct expression patterns of the prognostic genes in high-versus low-risk patients (Figures 5F,G). Moreover, immune checkpoint analysis revealed significant differences in expression patterns between high- and low-risk patients, with several key checkpoints strongly correlated with RSF-derived risk scores (Figure 5H; Supplementary Figure S4E). Predictive analysis of chemotherapy drug sensitivity reveals patients in the high-risk group exhibiting lower IC50 values for Camptothecin, suggesting they may derive greater benefit from this drug; conversely, patients in the low-risk group are more sensitive to Staurosporine. This difference in chemotherapy sensitivity based on NETosis-RGs risk stratification offers a framework for developing personalized therapy strategies for high-risk DLBCL patients, thereby possessing significant potential for clinical translation. For instance, in the management of high-risk patients, priority should be assigned to selecting chemotherapy drugs with lower IC50 values in the high-risk group, or investigating the combination of immune checkpoint inhibitors with specific chemotherapy drugs to overcome their immunosuppressive microenvironment and enhance efficacy. (Figures 5I,J). Next, we examined epigenetic regulation through m6A-related analyses. Heatmaps and boxplots revealed differential expression of m6A regulators between risk groups and highlighted their correlations with the five prognostic genes, thereby revealing potential differences in m6A modification patterns across these groups, suggesting that epigenetic regulation may contribute to the regulatory mechanisms of NETosis-RGs. (Supplementary Figures S4C,D). Notably, AKT1 and SLC25A37 exhibited the strongest associations with m^6^A regulatory proteins, which themselves displayed significant differential expression between risk groups, underscoring a potential epigenetic mechanism underlying RSF-based risk stratification. Finally, we constructed a speculative ceRNA regulatory network, providing a potential molecular framework for understanding the post-transcriptional regulation of these genes by identifying 200 miRNAs associated with the prognostic genes and 72 lncRNAs linked to 106 of these miRNAs. A gene–miRNA–lncRNA interaction network comprising 331 interactions was established, including notable examples such as FPR2-hsa-miR-640-ARIH2OS, AKT1-hsa-miR-6873-3p-DNAH10OS, and F3-hsa-miR-26b-5p-HHLA3. (Supplementary Figure S4F), revealing additional layers of regulatory control contributing to DLBCL progression. Collectively, these analyses provide a comprehensive view of the immune, epigenetic, and post-transcriptional regulatory landscapes associated with RSF-derived risk stratification, offering mechanistic insights into DLBCL progression and identifying potential avenues for targeted therapeutic interventions.

### 3.6 Single-cell transcriptomic analysis revealed extensive cellular heterogeneity and dynamic interactions within the immune microenvironment

**FIGURE 6:**
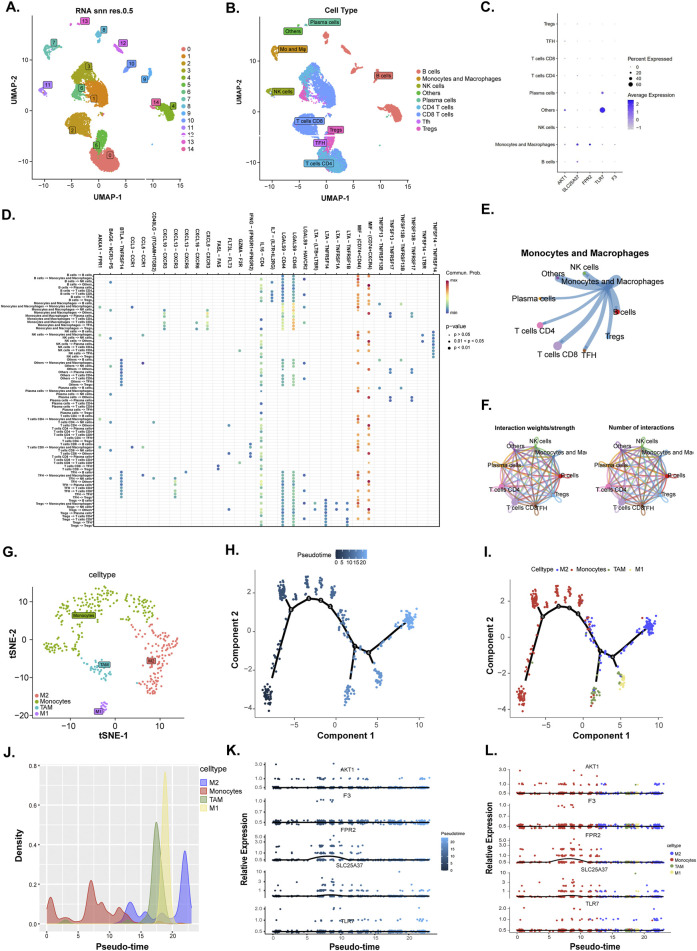
Single-cell transcriptomic landscape, intercellular communication, and pseudo-time dynamics of immune cell subsets in DLBCL. **(A)** UMAP plot depicting 15 transcriptionally distinct cell clusters identified in DLBCL. **(B)** UMAP plot annotated with immune cell subtypes. **(C)** Bubble plot showing the expression of prognostic NETosis-RGs across different cell clusters. **(D)** Bubble heatmap illustrating major signaling pathways mediating intercellular communication in DLBCL. **(E)** Monocytes/Macrophages-centered communication network highlighting their interactions with other cellular compartments. **(F)** Global intercellular interaction network depicting connectivity among cellular components in DLBCL. **(G)** t-SNE plot distinguishing monocytes, tumor-associated macrophages (TAMs), and M1/M2 macrophage subsets. **(H)** Feature gene selection for constructing pseudo-time trajectories of immune cells. **(I)** Inferred monocyte-to-macrophage differentiation trajectory. **(J)** Density plots putative the proposed temporal variability of monocyte/macrophage states. **(K,L)** Pseudo-time trajectories of immune cell subsets, visualized by pseudo-time **(K)** and by cell type **(L)**.

Single-cell transcriptomic analysis revealed extensive cellular heterogeneity and dynamic interactions within the immune microenvironment. To ensure data quality control, Single-cell sequencing data filtering was performed in GSE182434, resulting in the selection of 12,372 cells **(** Supplementary Figures S5A,B **)**. Data was normalized to control for quality, and after normalization, the gene expression counts more closely approximated a normal distribution **(** Supplementary Figures S5C,D **)**. The top 10 genes with the highest variability in expression between cells were identified and marked as C1QC, C1QB, C1QA, PTGDS, APOE, APOD, CDCL9, S100A8, IGLV2-23, and CCL4L2 **(** Supplementary Figure S5E **)**. Concurrently, a PCA scatter plot was constructed. Simultaneously, the top 2000 highly variable genes that were selected were normalized and subjected to PCA. The results showed that all samples were clustered in the spatial dimensions, with no outliers present **(** Supplementary Figures S5F-H). 15 transcriptionally distinct clusters (0–14) at a resolution of 0.5 were identified through UMAP projection, which were further annotated based on canonical marker genes, including B cells, Monocytes and Macrophages, NK cells, Plasma cells, T cells CD4, T cells CD8, TFH and Tregs (Figures 6A,B). The prognostic genes (AKT1, SLC25A37, FPR2, TLR7 and F3) exhibited relatively high expression levels in monocytes and macrophages (Figure 6C). To evaluate the intercellular communication, an analysis of cellular communication was performed. Through the bubble heatmap, it was observed that the communication probability values for MIF−(CD74+CXCR4) and MIF−(CD74^+^CD44) interactions were high (Figure 6D). Besides that, monocytes/macrophages had strong receptor-ligand interactions with a variety of other cell types, including T cells, plasma cells, NK cells, and myeloid populations, positioning them as key recipients and integrators of intercellular signals within the immune microenvironment. (Figure 6E; Supplementary Figure S5I). Global interaction networks further confirmed strong connectivity among B cells, T cells, NK cell and monocytes/macrophages (Figure 6F). To further investigate the role of monocytes/macrophages in DLBCL, monocytes and macrophages were distinguished based on the expression of the CD14 and CD16 (FCGR3A) genes, as well as the CD68 and HLA-DRA genes (Figure 6G; Supplementary Figures S5J-K). In order to further elucidate the role of monocytes/macrophages, highly variable genes in monocytes/macrophages were used as feature genes to construct a pseudo-time trajectory. This analysis infers a putative differentiation trajectory from monocytes to macrophages. Our analysis demonstrates that the expression of five prognostic genes exhibits dynamic regulation along this inferred trajectory. (Supplementary Figure S5L). The results showed that monocytes/macrophages progressively differentiated along a path from left to right (Figure 6H). During the early to middle stages of differentiation, the cells were monocytes, which gradually differentiated into M2 macrophages, TAM, and M1 macrophages (Figure 6I). In the initial phase of differentiation, the population was primarily composed of monocytes, which slowly progressed to produce a modest quantity of M2 macrophages and a subset of TAMs. As differentiation reached its terminal stage, there was a substantial increase in the presence of M2 macrophages (Figure 6J). And the relative expression levels of most prognostic genes were higher in monocytes. In addition to being highly expressed in monocytes, AKT1, SLC25A37, and TLR7 also showed an increase in expression in M1 macrophages at the end of differentiation (Figures 6K,L). Collectively, these analyses delineate the transcriptional heterogeneity, intercellular communication networks, and differentiation trajectories within the immune microenvironment, highlighting monocytes/macrophages as central integrators of NETosis signals of immune interactions and providing mechanistic insights into the regulation of myeloid cell states.

## 4 Discussion

This study provides an integrative multi-omics analysis of how NETosis-related molecular programs intersect with immune dysregulation in DLBCL. Rather than focusing solely on prognostic modeling, our work systematically links a NETosis-centered gene signature to the heterogeneity of the tumor immune microenvironment, the differentiation trajectories of myeloid cells, and the multi-layered regulatory networks that may govern these processes. Functional enrichment analysis placed the identified NETosis-RGs at the crossroads of innate immunity and inflammation—most notably within Toll-like receptor (TLR) signaling, AGE-RAGE signaling, and NET formation pathways. TLR7, one of the five core prognostic genes, activates immune cells and drives pro-inflammatory cytokine production upon recognizing single-stranded RNA (Hu et al., 2025; Mahla, 2024; Yang et al., 2024); the AGE-RAGE axis promotes tumor progression through pro-inflammatory cascades (Zhou et al., 2024); and the NET formation pathway itself embodies the functional duality of neutrophils in cancer (Wang et al., 2024; Wang et al., 2022). The convergence of these pathways on our gene signature suggests that NETosis-RGs may serve as sentinels of a broader immunoregulatory imbalance. This interpretation is reinforced by our single-cell data showing that the five prognostic genes are predominantly expressed in monocytes and M1 macrophages and exhibit dynamic regulation along myeloid/macrophage differentiation trajectories. These findings collectively delineate an immune architecture that differs fundamentally between risk strata and implies that NETosis activity may influence the balance between immunosuppressive and inflammatory states within the DLBCL microenvironment.

The immune cell composition diverged markedly between risk groups, reinforcing the notion that prognostic stratification based on NETosis-RGs captures genuine immunological heterogeneity. Naive B cells, which correlated positively with AKT1 (Pierau et al., 2012), were enriched in the low-risk group, consistent with a protective role through effective humoral immunity. Conversely, γδ T cells—potent tumor-recognizing effectors—were diminished in high-risk patients, likely reflecting tumor-immune evasion (Zhang et al., 2025; Arias-Badia et al., 2024). Notably, monocytes and M1 macrophages showed distinct association patterns with AKT1 and SLC25A37. Given that M1 polarization is linked to pro-inflammatory and anti-tumor activity (Ji et al., 2025), the low expression of these risk genes in M1 macrophages may facilitate a tumor-suppressive phenotype (Cai et al., 2012). These observations position monocytes/macrophages as central integrators of NETosis-related signals in the DLBCL immune microenvironment. Beyond transcript levels, our analyses uncovered additional regulatory dimensions that shape the immunological impact of NETosis-RGs. The observed differential m6A modification patterns and ceRNA networks, including the FPR2–hsa-miR-640–ARIH2OS and AKT1–hsa-miR-6873-3p–DNAH10OS axes, indicate potential epigenetic and post-transcriptional regulatory mechanisms. Similarly, distinct profiles of chemotherapy sensitivity and immune checkpoint expression across risk groups further underscore the potential clinical relevance of this immune-based risk stratification. However, as these findings are exploratory and hypothesis-generating, they necessitate further experimental and clinical validation. Our single-cell data demonstrate that five prognostic genes are highly expressed in monocytes and M1 macrophages, and they exhibit dynamic regulation across the inferred myeloid/macrophage differentiation trajectory.

Several limitations should be acknowledged. Our analyses relied on publicly available datasets, which may harbor inherent biases from variable sample sizes, patient demographics, and treatment protocols, potentially affecting generalizability. While we performed histological validation of NETosis markers (CitH3, NE, MPO) in clinical DLBCL specimens, direct functional perturbation of the identified prognostic genes was not undertaken; establishing causal roles for AKT1, SLC25A37, FPR2, TLR7, and F3 in NETosis-dependent immune modulation will require future *in vitro* and *in vivo* studies. Additionally, the full resolution of cellular heterogeneity in the immune microenvironment remains constrained by current single-cell technologies. Prospective validation in multi-center cohorts will be essential to translate this multi-omics immune signature into clinical practice.

In conclusion, by integrating bulk and single-cell transcriptomics with machine learning, this study defines a NETosis-centered immune signature that robustly stratifies DLBCL patients and illuminates the cellular and molecular underpinnings of immune dysregulation in this disease. The convergence of prognostic gene expression on monocytes and M1 macrophages, the dynamic regulation along myeloid differentiation trajectories, and the surrounding network of m^6^A modifications and ceRNA interactions collectively provide a mechanistic framework for future immunomodulatory therapeutic strategies. These findings advance our understanding of DLBCL not merely as a malignancy of B-cell origin but as a disease whose clinical trajectory is significantly influenced by the innate immune compartment.

## Data Availability

The datasets presented in this study can be found in online repositories. The names of the repository/repositories and accession number(s) can be found in the article/Supplementary Material.
