# Supplementary material for: Decoding NETosis-associated immune dysregulation in diffuse large B-cell lymphoma through integrative multi-omics and machine learning
Source: Front Cell Dev Biol. 2026 Jul 2;14:1867660. doi: 10.3389/fcell.2026.1867660 (PMC13372773; doi:10.3389/fcell.2026.1867660)
Supplement: Supplementary file 1 [file DataSheet1.DOCX]

**
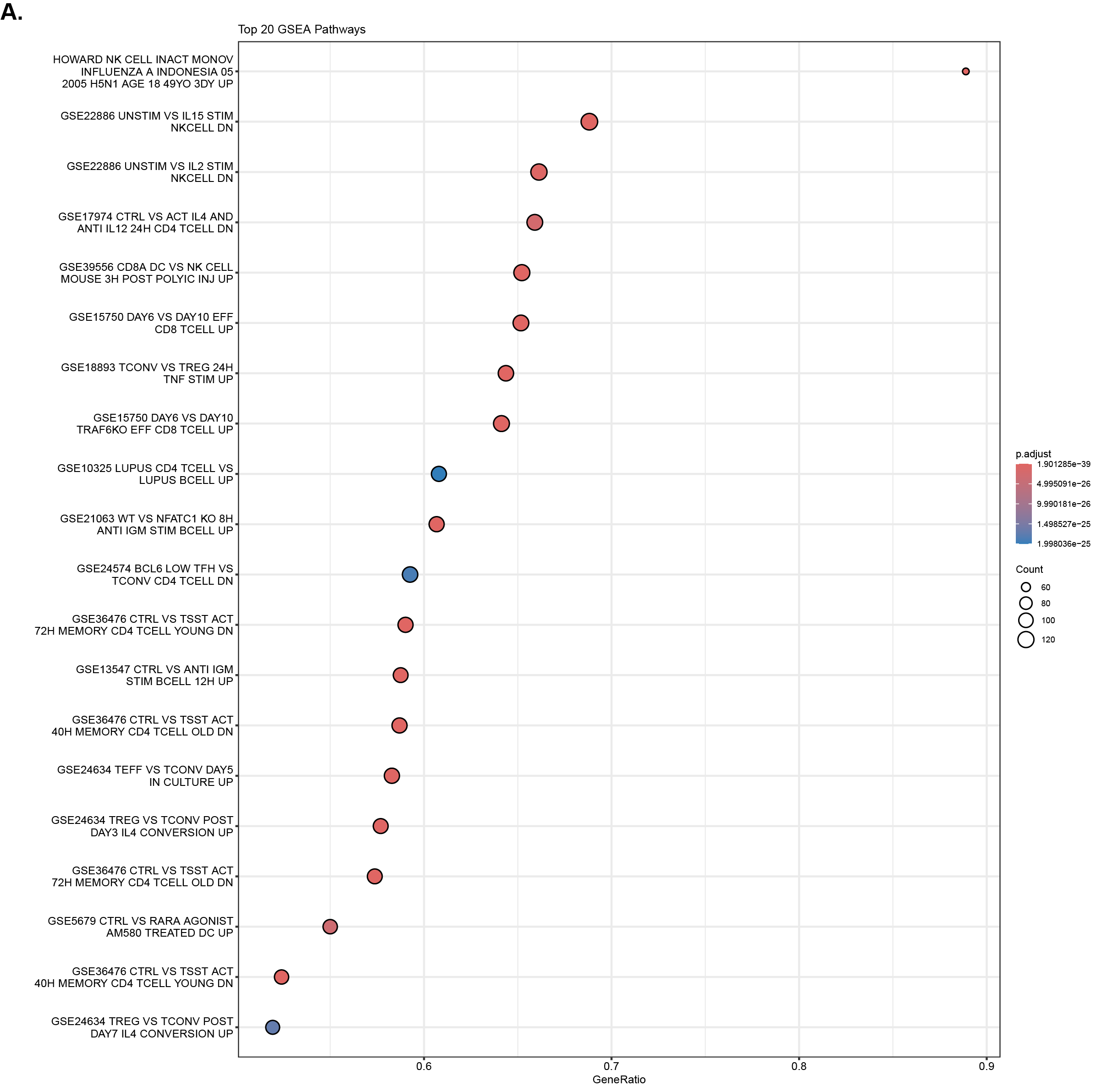
Supplementary Figure Legends**

**Figure S1. Top 20 enriched pathways identified by GSEA analysis.** (**A**)Dot plot showing the top 20 significantly enriched pathways ranked by normalized enrichment score (NES) in the gene set enrichment analysis (GSEA).


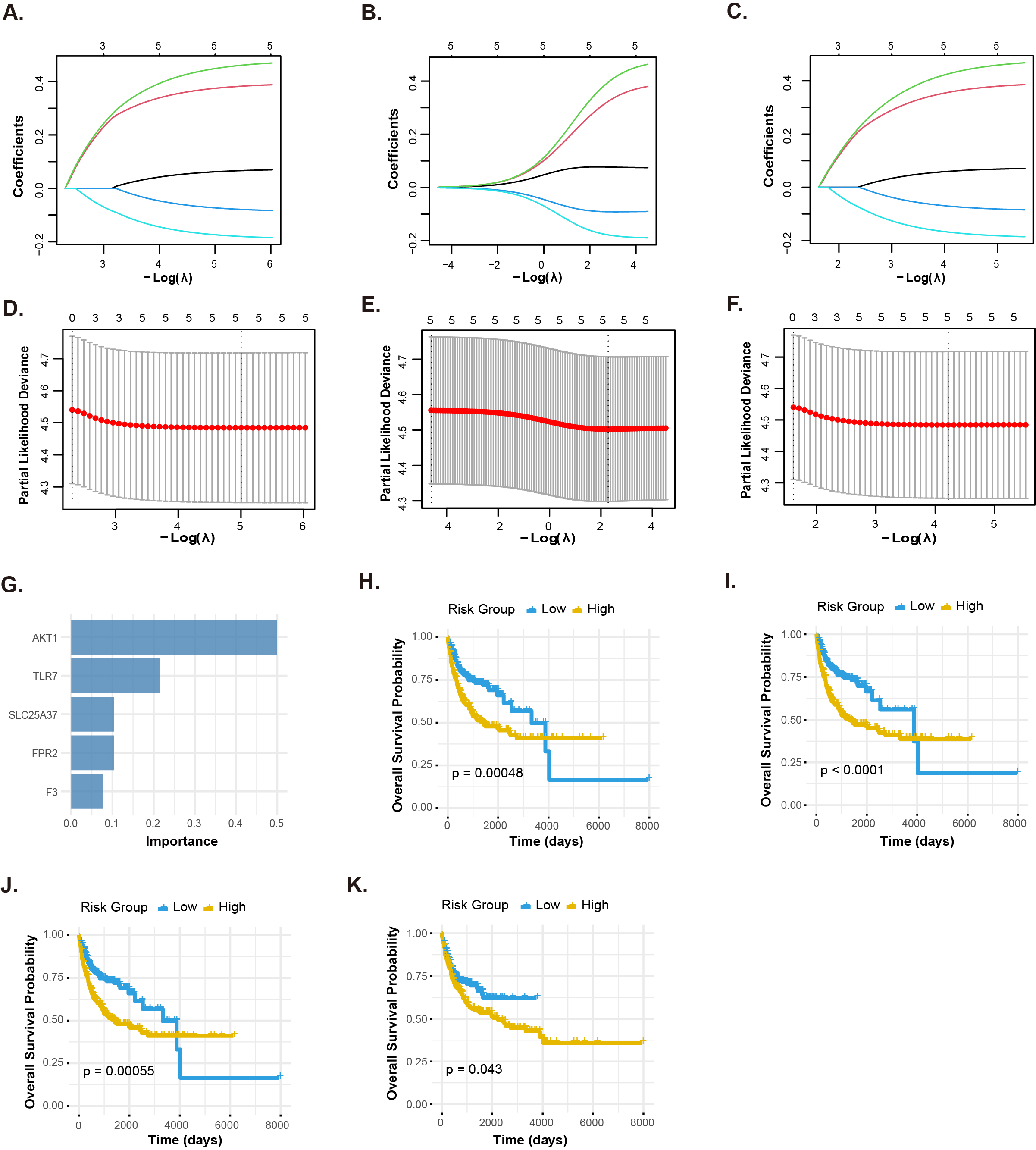


**Figure S2. Penalized regression and XGBoost modeling of five prognostic NETosis-RGs: tuning, feature importance, and survival analysis.** (**A-C**) Coefficient trajectories for LASSO (A), Ridge (B), and Elastic Net (C) regression models. The x-axis represents −log(λ), and the y-axis shows the estimated regression coefficients for each prognostic gene. Vertical dotted lines indicate the λ value selected by cross-validation. (**D-F**) Partial likelihood deviance curves for LASSO (D), Ridge (E), and Elastic Net (F) models. The x-axis represents −log(λ), and the y-axis shows the partial likelihood deviance. The minimum deviance indicates the optimal λ.(**G**) XGBoost feature importance plot showing relative contribution of each prognostic gene to model performance. (**H–K**) Kaplan–Meier survival curves for patients stratified into high- and low-risk groups based on LASSO (H), Ridge (I), Elastic net (J), and XGBoost (K) models. Survival differences were assessed by log-rank test.


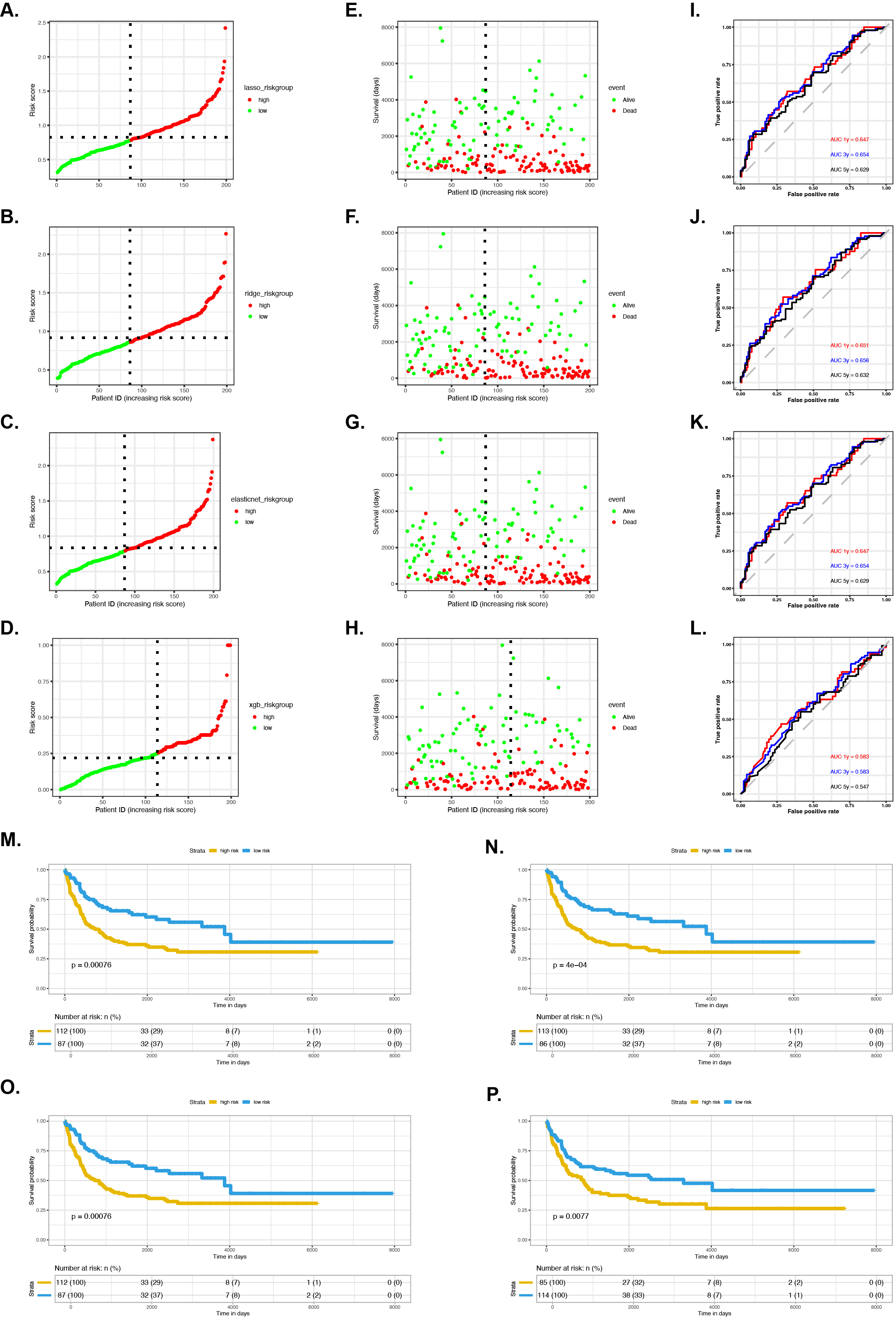
**Figure S3. External validation of multiple prognostic NETosis-RG models in DLBCL patients.** (**A-D**) Distribution of risk scores for patients in LASSO (A), Ridge (B), Elastic Net (C), and XGBoost (D) models, ranked by increasing risk score. High-risk and low-risk groups are indicated. (**E-H**) Corresponding survival status and days for each patient in the models shown in (A–D). Shorter survival times are predominantly observed in the high-risk groups. (**I-L**) Time-dependent receiver operating characteristic (ROC) curves assessing predictive performance of LASSO (I), Ridge (J), Elastic Net (K), and XGBoost (L) models at 1-, 3-, and 5-year overall survival. (**M-P**) Kaplan–Meier survival curves stratified by risk scores from LASSO (M), Ridge (N), Elastic Net (O), and XGBoost (P) models. Numbers at risk for high- and low-risk groups are shown below each curve.


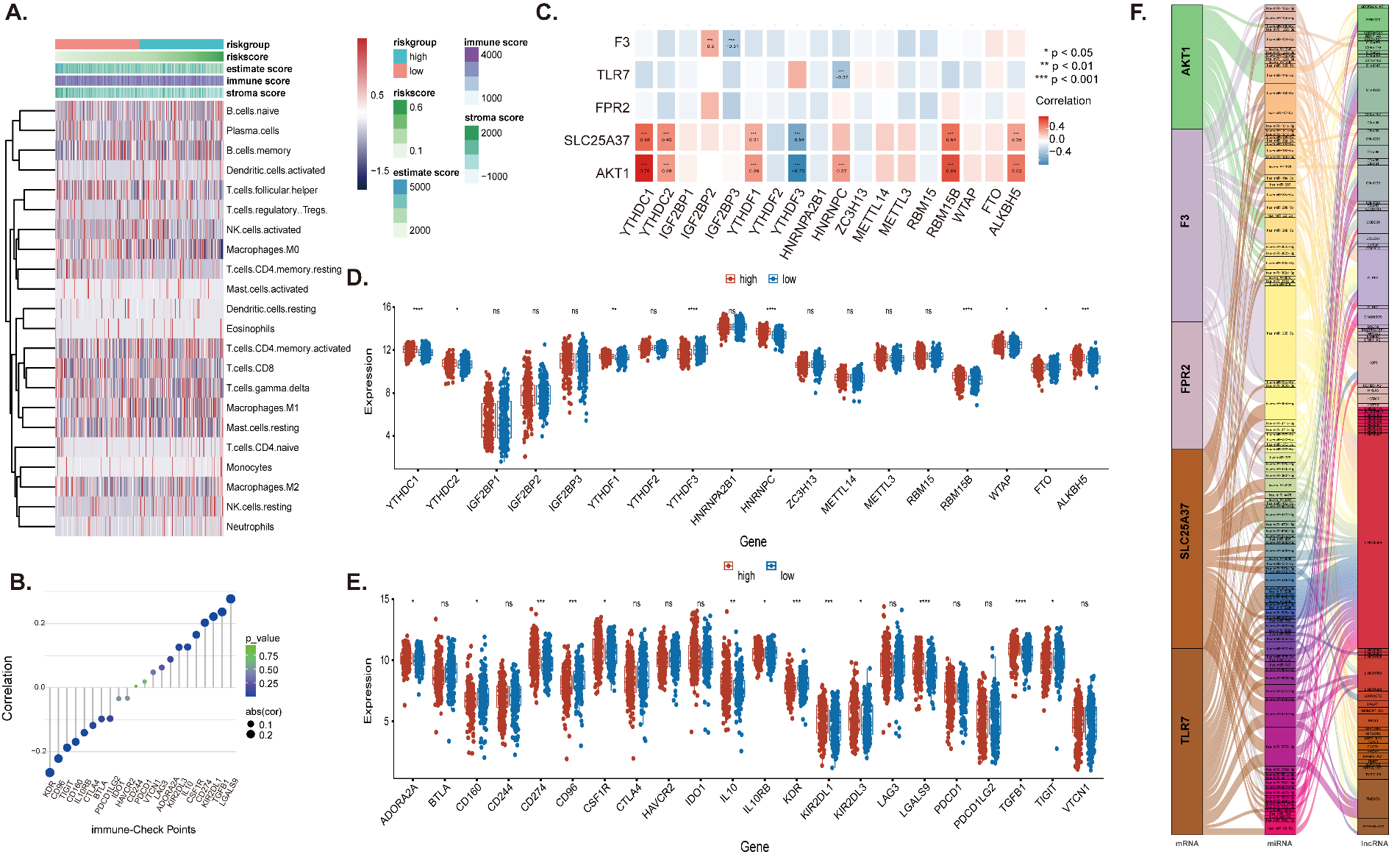
**Figure S4. Immune, epigenetic, and ceRNA network characterization of prognostic NETosis-RGs in DLBCL.** (**A**) Heatmap clustering showing the relationship between RSF-derived risk scores, risk groups, and immune cell types across patients. (**B**) Lollipop plot comparing proportions of immune cell types between high- and low-risk groups. (**C**) Heatmap showing expression of m6A-related proteins and the five prognostic NETosis-RGs. (**D**) Dot plot comparing m6A protein expression between high- and low-risk groups. (**E**) Dot plot of immune checkpoint gene expression between high- and low-risk groups. (**F**) ceRNA network connecting lncRNAs, miRNAs, and the five prognostic NETosis-RGs by Sankey Diagram.


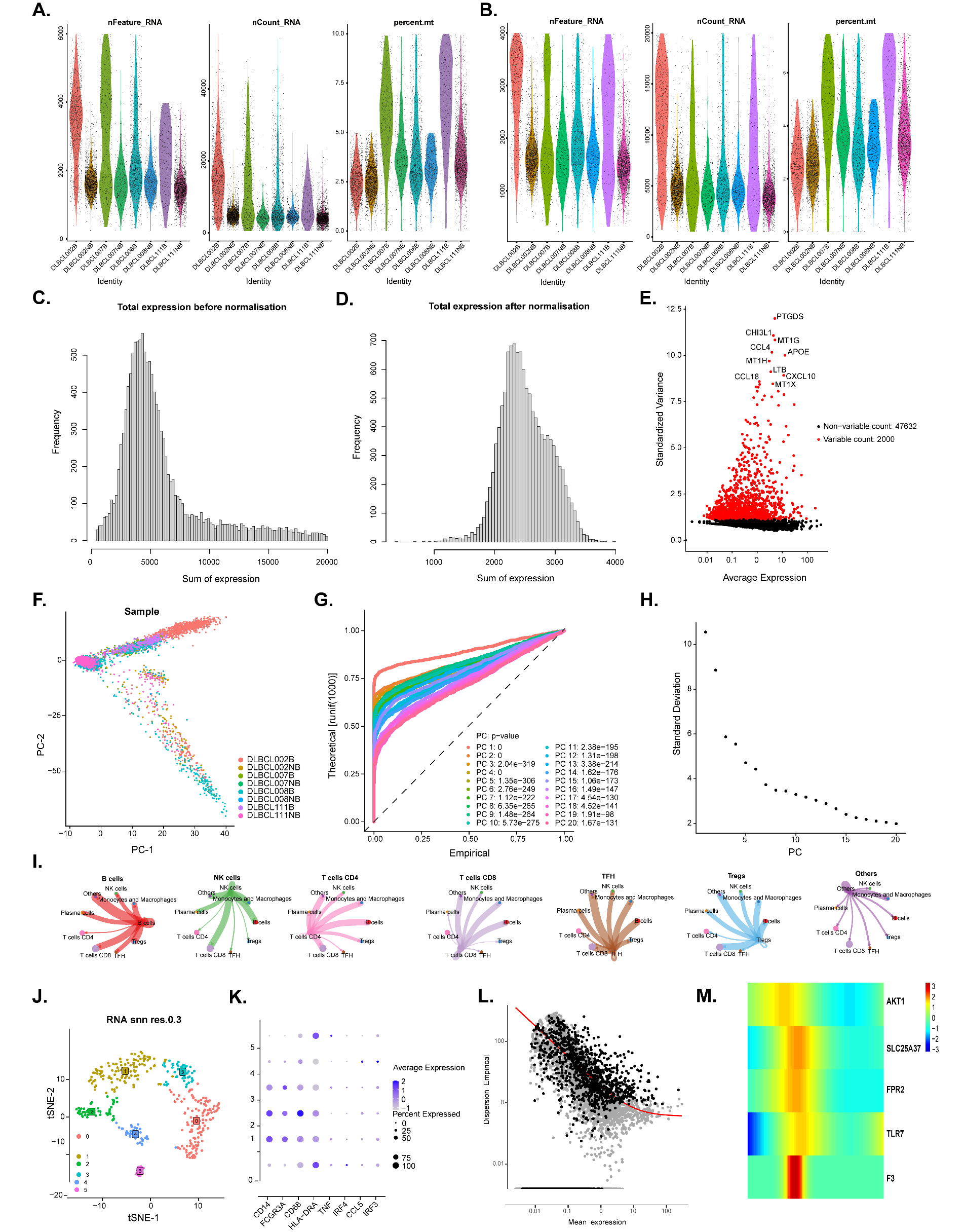


**Figure S5. Quality control, dimensionality reduction, and single-cell analyses of DLBCL.** (**A-B**) Violin plots showing quality control metrics of single-cell sequencing data before (A) and after (B) filtering. (**C-D**) Gene expression distributions before (C) and after (D) normalization. (**E**) Scatterplot of highly variable genes across cells. (**F**) PCA plot of the top 2000 highly variable genes. (**G-H**) JackStraw and ScoreJackStraw plots illustrating PCA inflection points and variance explained for dimensionality reduction. (**I**) Cell–cell communication network map showing interactions among cellular components in DLBCL. (**J**) t-SNE plot identifying six distinct cell clusters. (**K**) t-SNE plot showing annotation of monocyte and macrophage subtypes based on canonical biomarkers. (**L**) Scatterplot of pseudo-time trajectories in monocytes/macrophages based on highly variable genes. (**M**) Heatmap displaying temporal dynamics of prognostic genes across pseudo-time.
